# The Changing Landscape in Patients With Crohn's Disease

**DOI:** 10.1002/ueg2.70172

**Published:** 2026-01-09

**Authors:** Solomon Ong, John Paul Seenan

**Affiliations:** ^1^ Queen Elizabeth University Hospital Glasgow UK

## 1 Introduction

The demographics of Crohn’s disease (CD) are shifting with increasing cases among older adults [1]. This is driven by the natural aging of our existing CD cohort and an increasing incidence of elderly‐onset CD. Despite this, there are limited studies focusing on elderly‐onset CD. Older patients remain under‐represented in clinical research as they are often excluded from trials due to age, comorbidities or polypharmacy. This has led to uncertainty in the management of elderly‐onset CD. When an older patient with Crohn’s disease undergoes intestinal resection, the clinician therefore faces a difficult dilemma—whether to start immunosuppressive therapy to prevent recurrence or withhold treatment to minimise risk of infection but risk the recurrence of disease.

## 2 Crohn's Disease in the Elderly

In this study using the ENEIDA registry, Mañosa et al. provide a crucial insight into this dilemma. Their work represents the largest study to date evaluating postoperative outcomes in older patients with CD. It provides some interesting insights and poses important questions for those of us involved in managing these patients [2].

Their findings challenge the theory that elderly onset CD is inherently benign or burnt out due to immunosenescence [3]. The study showed that the elderly patients had a different disease behaviour and course of progression, there are fewer penetrating complications and perianal disease but more stricturing behaviour.

Balancing the risks and benefits of post‐operative prevention in Crohn's disease can be difficult. This is particularly true in elderly patients when the risks of medical treatment (e.g., risks of malignancy with thiopurines [4] or sepsis with anti‐TNF medications [5] are known to be higher. Despite elderly patients receiving significantly less preventive therapy than the controls, the rate of postoperative recurrence were similar. The cumulative risk of needing a second resection was approximately 10% at 10 years for the elderly patients.

While a 10% recurrence rate might seem manageable, the physiological cost of a second major abdominal surgery in an elderly patient is often much higher than in a young patient. The study noted that perioperative mortality was double in the elderly group. Hence, the ‘watch and wait’ strategy of withholding treatment to ensure safety may paradoxically expose elderly patients to the life‐threatening risks of repeat intervention.

For a frail patient with limited life expectancy, a 10‐year recurrence risk of 10% may be clinically acceptable to avoid any immediate risks, potential side‐effects or inconvenience of medical therapy. However, conversely, for a fit older patient with a longer life expectancy, this same risk may justify the use of preventative therapy to avoid the increased mortality risk and additional morbidity of repeat resection.

## 3 The Thiopurine Paradox

Interestingly, the study found that only thiopurines were independently associated with a reduced risk of surgical recurrence. This finding reflects the study period (2005–2020), where newer treatments with more favourable safety profiles like anti‐integrins (vedolizumab), p40 inhibitors (ustekinumab) and p19 inhibitors (risankizumab, mirikizumab and guselkumab) were unavailable or underutilised. Many clinicians have moved away from using thiopurines in older patients due to concerns about lymphoma and non‐melanoma skin cancer [6]. Yet, in this real‐word cohort, it shows that thiopurines may still have a role in the elderly IBD cohort.

## 4 The Monitoring Dilemma: Surgical, Endoscopic, and Biochemical Recurrence

The POCER study established the gold standard for post‐operative care is to perform a colonoscopy 6–12 months after surgery [7]. However, applying is this standard to the elderly patient can be difficult as bowel preparation may be poorly tolerated and the sedation carries higher cardiopulmonary risks. Consequently, there is often a greater reliance on clinical symptoms or biochemical markers (C‐reactive protein or faecal calprotectin). However, these surrogates are not as accurate and reliable as endoscopic monitoring. This study could not capture endoscopic or biochemical recurrence rates due to its retrospective nature. As a result, the true risk of post‐operative recurrence in older patients remains unclear and could be even greater if, as may be expected, the threshold for repeat resection in this group is higher.

## 5 Conclusion

This study demonstrates the strengths of using registry data to help inform decision‐making in patient sub‐groups but also highlights the limitations of a retrospective study such as the potential for selection bias.

This emphasises the need for prospective data collection in elderly‐onset CD. Future studies should aim to delineate the natural history, define the role of frailty scores as well as validate the accuracy of non‐invasive monitoring and patient reported outcomes in this cohort. Until them, the management plan in elderly‐onset CD should be an active decision based on frailty and risk, not a default based on age.

## Conflicts of Interest

The authors declare no conflicts of interest.

## Data Availability

The authors have nothing to report.
